# Gene Expression Value Prediction Based on XGBoost Algorithm

**DOI:** 10.3389/fgene.2019.01077

**Published:** 2019-11-12

**Authors:** Wei Li, Yanbin Yin, Xiongwen Quan, Han Zhang

**Affiliations:** ^1^College of Artificial Intelligence, Nankai University, Tianjin, China; ^2^Department of Food Science and Technology, University of Nebraska–Lincoln, Lincoln, NE, United States; ^3^Key Laboratory for Medical Data Analysis and Statistical Research of Tianjin, Nankai University, Tianjin, China

**Keywords:** gene expression value, landmark gene, target gene, regression method, XGBoost, absolute error

## Abstract

Gene expression profiling has been widely used to characterize cell status to reflect the health of the body, to diagnose genetic diseases, etc. In recent years, although the cost of genome-wide expression profiling is gradually decreasing, the cost of collecting expression profiles for thousands of genes is still very high. Considering gene expressions are usually highly correlated in humans, the expression values of the remaining target genes can be predicted by analyzing the values of 943 landmark genes. Hence, we designed an algorithm for predicting gene expression values based on XGBoost, which integrates multiple tree models and has stronger interpretability. We tested the performance of XGBoost model on the GEO dataset and RNA-seq dataset and compared the result with other existing models. Experiments showed that the XGBoost model achieved a significantly lower overall error than the existing D-GEX algorithm, linear regression, and KNN methods. In conclusion, the XGBoost algorithm outperforms existing models and will be a significant contribution to the toolbox for gene expression value prediction.

## Introduction

Characterizing gene expression patterns in cells under various conditions is an important problem (Aigner et al., 2010). Gene expression profiling is a vital biological tool commonly used to capture the response of cells to disease or drug treatments (Celis et al., 2000; Mclachlan et al., 2005; Wang et al., 2006; Mallick et al., 2009; Zeng et al., 2016). Although the cost of gene expression profiling is steadily decreasing in recent years, it is still very expensive when dozens or hundreds of samples need to processed (Chen et al., 2016).

Genes expression are closely related, and some methods for gene co-expression have also been extensively studied in recent years to further explore the relationship between gene expression. (Ozerov et al., 2016; Borisov et al., 2019). Considering that gene expressions are usually highly correlated, researchers conducted an in-depth analysis of gene expression profiles and found that ∼1,000 genes can capture about 80% of the entire gene expression profile (Lamb et al., 2006). These genes are called landmark genes, and the remaining genes are called target genes (Penfold and Wild 2011). Inspired by this, many scholars have suggested that the expression value of the landmark gene can be used to predict the expression value of the target gene, which will greatly reduce the cost of the gene expression profiling (Chen et al., 2016). The cost of measuring expression profiles containing only ∼1,000 landmark genes will be much lower, compared with profiles across the whole human genome. If researchers want to study the expression of a particular target gene, it can be inferred by the landmark genes.

However, this task is very difficult because, in principle, gene expression value prediction is a multi-task regression problem. In 2016, Yifei Chen et al. proposed the D-GEX algorithm based on Back Propagation neural network (Chen et al., 2016), in which 943 landmark genes correspond to 943 input units, and 9,520 target genes correspond to 9,520 output units. However, the prediction accuracy of this algorithm still has a large room for improvement. Besides, deep network has poor interpretability, and for each target gene, we cannot know which landmark genes have much greater impact on its expression. Last but not the least, deep network needs to read all the data into the memory at the time of training, and therefore, the algorithm is prone to occupy excessive memory in actual use, and has high demand for GPU too.

In addition to deep network, some researchers also used linear regression, KNN and other classical algorithms for target gene expression prediction (Chen, 2014), but the prediction results of these algorithms were less accurate.

Among the Boosting Tree models, XGBoost (Chen and Guestrin, 2016) has a very strong expansion and flexibility. It integrates multiple tree models to build a stronger learner model. Furthermore, XGBoost is characterized by its ability to automatically use the multithreading of the CPU for parallel computing, which can speed up the calculation.

Based on the above research background, we proposed a new gene expression value prediction algorithm based on XGBoost, and established a regression prediction model for each target gene independently. The results showed that the XGBoost algorithm significantly improved the prediction accuracy, which is superior to D-GEX, LR, KNN, and other algorithms. It also had better predictive ability and generalization ability. Lastly, the XGBoost algorithm had stronger interpretability than other algorithms.

## Materials and Methods

In this section, we first introduced the dataset we used for this task. Then, we gave an introduction of XGBoost algorithm, and finally, we showed three competing methods.

### Dataset

The dataset used in this paper is the same as the dataset used by Yifei Chen et al. in the proposed D-GEX algorithm in 2016, which is the GEO (Gene Expression Omnibus, GEO) dataset selected by the Broad Institute from the published gene expression database (Edgar et al., 2008), and the RNA-Seq expression data which was from the Genotype-Tissue Expression (GTEx) project (Lonsdale et al., 2013; GTEx Consortium, 2015). In both dataset, each of sample has 943 landmark genes and 9,520 target genes after pre-processing.

**Figure 1:**
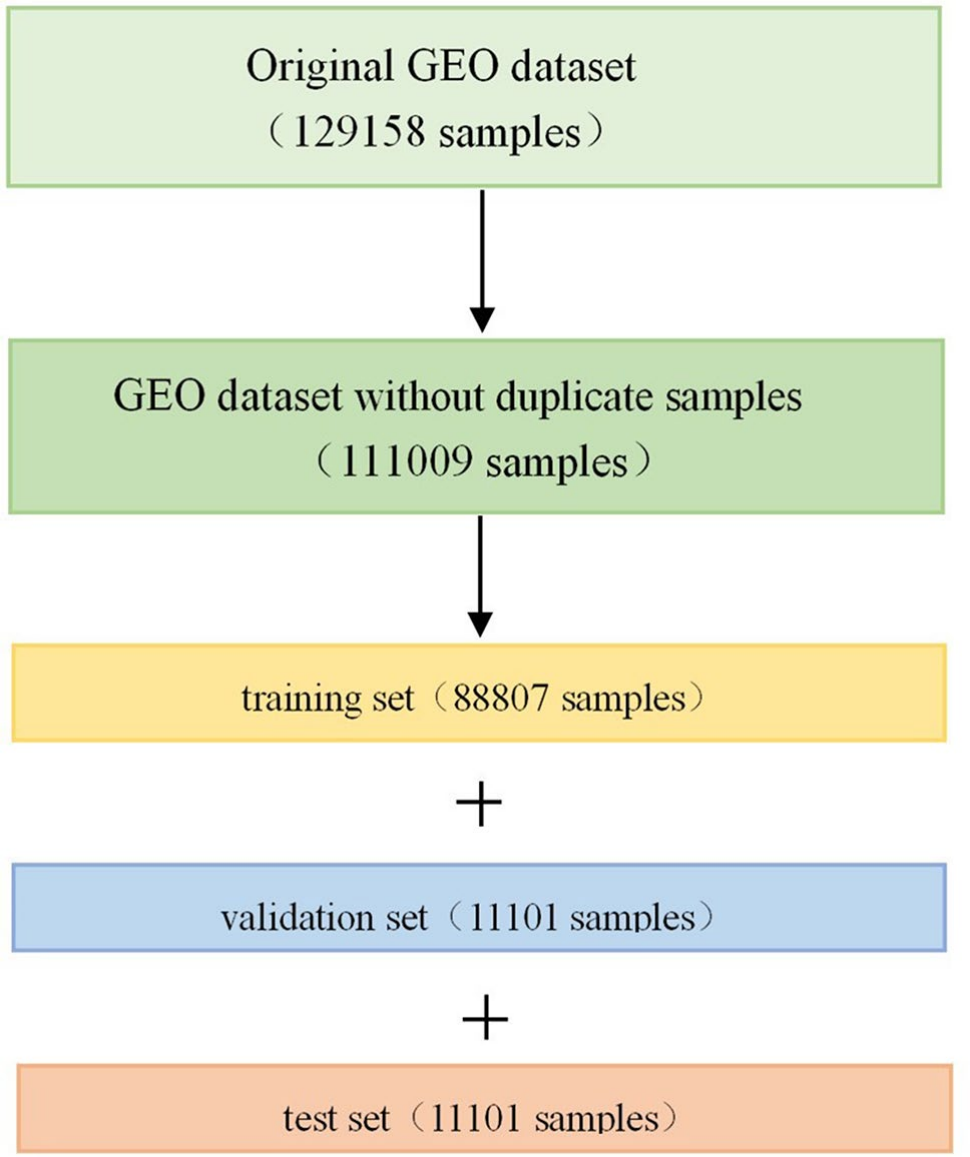
Division of Gene Expression Omnibus (GEO) dataset. Firstly, we removed the duplicate samples from the original GEO dataset, and then divided it into training set, validation set, and test set in a scale of 8:1:1 after randomly shuffling.

The GEO dataset has a total of 129,158 gene expression profiles of cell line samples, and it should be noted that we refer to each profile as a sample in this article. The original GEO dataset was generated by the Affymetrix microarray platform, and the expression values are in a numerical range between 4 and 15. Since some of the samples are repetitive or highly similar, we first removed the duplicate samples from the 129,158 samples in order to avoid unnecessary calculations. All samples were clustered into 100 classes using the k-means algorithm (Hartigan and Wong 1979; Chen et al., 2016). In each class, the pairwise Euclidean distance between the two samples was calculated. If the pairwise Euclidean distance was less than 1.0, one of the samples was removed. After removing the duplicate samples, 111,009 samples were obtained, which were divided into training set, validation set and test set according to the ratio of 8:1:1 after randomly shuffling (Figure 1). Therefore, there were 88,807 samples in the training set, 11,101 samples in the validation set, and 11,101 samples in the test set.

We used the training set to train the models, and adjusted the parameters based on the performance on the validation set. Finally, we used the results on the test set to evaluate the model.

We also performed experiments on RNA-Seq expression data to further evaluate the reliability of the model. The RNA-Seq expression data includes GTEx expression data and 1,000 Genomes expression data (1,000G). The GTEx expression data consist of 2921 profiles, which were obtained from various tissue samples (GTEx Consortium, 2015), and the 1,000G expression data have 462 profiles of lymphoblastoid cell line samples (Lappalainen et al., 2013). They were both obtained from the Illumina RNA-Seq platform and measured based on Gencode V12 annotations (Lappalainen et al., 2013; GTEx Consortium, 2015).

Like Chen et al. designed before, we still used the training set of the GEO dataset as the training set, then used 1,000G data as the validation set, and finally employed GTEx dataset as the test set to further evaluate the generalization ability of the models based on this cross-platform experiment (Chen et al., 2016).

However, the GEO dataset and the RNA-seq dataset were obtained from different platforms, so the numerical scales were different as well. Therefore, we performed quantile normalization on all the datasets, which means that all the datasets were standardized by subtracting the mean and then dividing by the standard deviation of each gene (Chen et al., 2016).

### XGBoost Algorithm

XGBoost (Extreme Gradient Boosting) is a model that was first proposed by Tianqi Chen and Carlos Guestrin in 2011 and has been continuously optimized and improved in the follow-up study of many scientists (Chen and Guestrin, 2016). The model is a learning framework based on Boosting Tree models.

The traditional Boosting Tree models uses only the first derivative information. When training the *n_th_* tree, it is difficult to implement distributed training because the residual of the former *n-1* trees is used. XGBoost performs a second-order Taylor expansion on the loss function and it can automatically use the multithreading of the CPU for parallel computing. Besides, XGBoost uses a variety of methods to avoid overfitting.

The XGBoost algorithm is briefly introduced as follows (Chen and Guestrin, 2016), and the details are given in the **Supplementary Material**.

Integrate the tree model with addition method, assuming a total of *K* trees, and use *F* to represent the basic tree model, then:

(1) $${\hat{y}}_{i}=\sum_{k=1}^{K}f_{k}(x_{i}),f_{k}\in F$$

The objective function is:

(2) $$L=\sum_{i}l({\hat{y}}_{i},y_{i})+\sum_{k}\Omega (f_{k})$$

where *l* is the loss function, which represents the error between the predictive value and the true value; Ω is the function used for regularization to prevent overfitting:

(3) $$\Omega (f)=\gamma T+\frac{1}{2}\lambda ∥w{∥}^{2}$$

where *T* represents the number of leaves per tree, and *w* represents the weight of the leaves of each tree.

After the second-order Taylor expansion of the objective function and other calculations which are detailed in **Supplementary Material**, we can finally get the information gain of the objective function after each split is:

(4) $$\mathrm{Gain}=\frac{1}{2}\left[\frac{(\sum_{i\in I_{L}}g_{i}{)}^{2}}{\sum_{i\in I_{L}}h_{i}+\lambda }+\frac{(\sum_{i\in I_{R}}g_{i}{)}^{2}}{\sum_{i\in I_{R}}h_{i}+\lambda }+\frac{(\sum_{i\in I}g_{i}{)}^{2}}{\sum_{i\in I}h_{i}+\lambda }\right]-\gamma$$

As can be seen from (4), in order to suppress the growth of the tree and prevent the model from overfitting, a splitting threshold *γ* is added. The leaf node is allowed to split if and only if the information gain is greater than *γ*. This is equivalent to pre-pricing the tree while optimizing the objective function.

In addition, we also used the following two excellent techniques of XGBoost to avoid overfitting in the experiment:

1. If all sample weights on the leaf nodes are less than the threshold, the splitting is stopped. This prevents the model from learning special training samples.
2. Sample features randomly when building each tree.

These methods all make XGBoost more generalizable and get better performance in practical applications.

In the experiment, the regression model based on XGBoost was independently trained for each target gene, and the number of input landmark genes was 943, which means the input feature dimension was 943, and this dimension is very high. However, many techniques in XGBoost for avoiding overfitting can help reduce the degree of overfitting and improve the accuracy of regression prediction.

When the XGBoost model was actually used in the experiment, the following parameters were adjusted to make the model perform its best performance:

1. n_estimators
  *n_estimators* is the number of iterations in training. A too small *n_estimators* can lead to underfitting, which makes the model not fully perform its learning ability. However, a too large *n_estimators* is usually not good either, because it will cause overfitting.
2. min_child_weight
  As we mentioned earlier, *min_child_weight* defines the sum of sample weight of the smallest leaf nodes to prevent overfitting.
3. max_depth
  It is the maximum depth of the tree. The greater the depth of the tree, the more complex the tree model is, and the stronger the fitting ability is, but at the same time, the model is much easier to overfit.
4. subsample
  This parameter means the sampling rate of all training samples.
5. colsample_bytree
  The last parameter that we need to config is *colsample_bytree*. It is the feature sampling rate when constructing each tree. In this task, this is equivalent to the sampling rate of the landmark gene.
6. learning_rate
  In most algorithms, *learning rate* is a very important parameter that needs to adjust, as well as in XGBoost. It greatly affects the performance of the model. We can reduce the weight of each step to make the model more robust.

The details of parameters configuration were introduced in Section 3.

### Other Existing Methods

There are other methods that researchers have previously proposed that could be used in the gene expression value prediction task. In this section, we briefly describe these methods, and in next section, we evaluate the performance of XGBoost model by comparing the predictive results of XGBoost model with results of these existing models.

#### D-GEX

D-GEX (Chen et al., 2016) is the algorithm proposed by Yifei Chen and other researchers in 2016, which uses the classical BP neural network model. The number of landmark genes is 943 and the number of target genes is 9,520, so theoretically the number of input and output neurons of the network is 943 and 9,520, respectively. However, in actual training, Yifei Chen et al. randomly divided 9,520 target genes into two groups due to GPU memory limitation, and each group contained 4,760 target genes. Therefore, the network was also divided into two independent networks, corresponding to 943 input neurons and 4,760 output neurons, and trained independently on two GPUs.

Besides, the network used mean square error as the loss function:

(5) $$L_{\mathrm{BP}}=\sum_{t=1}^{T}\left[\frac{1}{N}\sum_{i=1}^{N}{\left(y_{i}(t)-{\hat{y}}_{i}(t)\right)}^{2}\right]$$

where *T* was the number of target genes and *N* was the number of training samples. The D-GEX algorithm selected one, two, or three hidden layers, respectively. The number of neurons in each hidden layer of the network was all the same, 3,000, 6,000, or 9,000,respectively. In addition, they added Dropout Layer (Srivastava et al., 2014) to the network to reduce the overfitting, and Momentum Method (Sutskever et al., 2013) was used to accelerate training, making the model approach the optimal much faster.

#### Linear Regression

A linear regression model was independently established for each target gene t as follows (Chen et al., 2016):

(6) $$f_{\left(t\right)}(x)=w_{\left(t\right)}^{T}x+b_{\left(t\right)}$$

where *w_(t)_* and *b_t_* can be calculated by the following formula:

(7) $$(w_{\left(t\right)},b_{\left(t\right)})=\underset{w,b}{\arg\min}\frac{1}{N}\sum_{i=1}^{N}{\left(y_{i(t)}-w_{\left(t\right)}^{T}x_{i}-b_{\left(t\right)}\right)}^{2}$$

On the basis of (16), by adding the L1 or L2 regularization term, the LR-L1 model and LR-L2 model can be obtained.

#### KNN

KNN is a non-parametric learning algorithm. For each target gene, the training samples were used to calculate the Euclidean distance of this target gene to all the landmark genes during training, and the k landmark genes with the smallest Euclidean distance were determined as the k-nearest neighbor landmark genes of the target gene (Hartigan and Wong, 1979; Chen et al., 2016). The average of the expression values of the k-nearest neighbor landmark genes of the target gene will be used as the predictive value.

The range of k value we tried in the experiment were integers between 2 and 20. We found that when the k value changed from 2 to 5, the prediction error was gradually decreasing; and from 5 to 20, the error was gradually increasing. Therefore, the optimal k value we found in the KNN model is 5.

## Results

In this section, we firstly introduced the process of parameters configuration of XGBoost algorithm and its high interpretability. Then, we showed the results of XGBoost model on both the GEO data and the GTEx data, and compared it with the previous methods.

### Tuning Model Parameters

*GridSearchCV*, a sub-module of the *sklearn* module in Python (Pedregosa et al., 2011), was used in the experiment to conduct grid search on all parameters to find the optimal parameters. The details of the tuning parameters are shown in **Table 1**:

**Table 1:** Detailed parameters configuration.

Parameters	Initialization value	Search space
*n_estimators*	300	[300, 330, 350, 370, 400]
*γ*	0	[0, 0.1, 0.2, 0.3, 0.4]
*min_child_weight*	1	[1, 2, 3, 4, 5, 6]
*max_depth*	5	[6, 7, 8, 9, 10, 11]
*subsample*	0.6	[0.6, 0.7, 0.8, 0.9]
*colsample_bytree*	0.8	[0.6, 0.7, 0.8, 0.9]
*learning_rate*	0.1	[0.01, 0.05, 0.08, 0.1]

Take the target gene CHAD for example, we established its XGBoost regression model. We initialized all the parameters of the model as shown in the above **Table 1**, and adjusted them in order.

**Figure 2:**
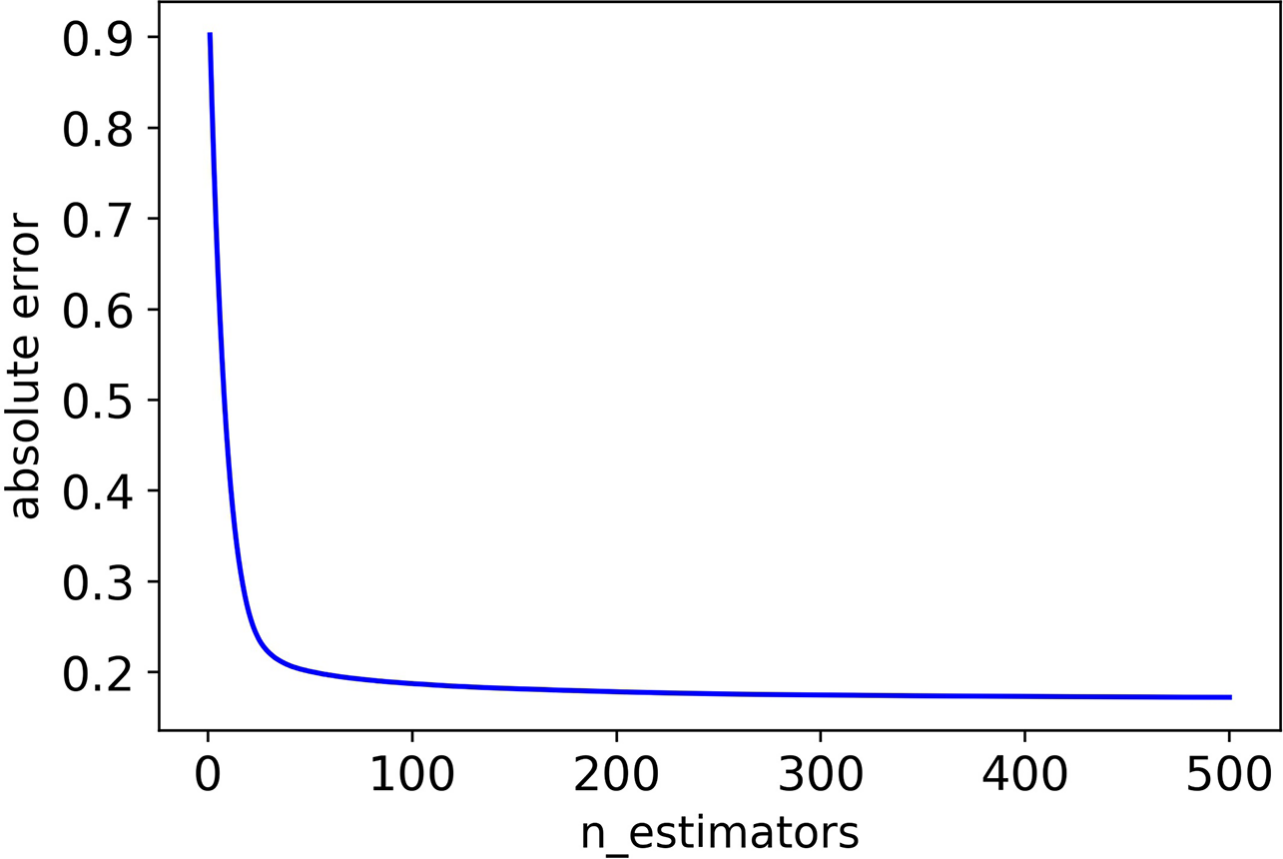
The absolute error of CHAD validation set decreases as *n_estimators* increases.

Firstly, we adjusted *n_estimators*, and the absolute error of CHAD gene changes with *n_estimators* as shown in **Figure 2** below:

It can be seen that the absolute error of the validation set did not decrease after 350 iterations, and in order to prevent overfitting, the optimal value of *n_estimators* was set as 350.

Update the value of *n_estimators* to 350 and adjust the next parameter γ, **Table 2** shows the absolute error of validation set corresponding to different γ values.

**Table 2:** Absolute errors of validation set corresponding to different γ.

γ	Absolute error
0	0.1712
0.1	**0.1701**
0.2	0.1709
0.3	0.1718
0.4	0.1709
0.5	0.1714

The figure in bold represents the lowest absolute error.

As can be seen from **Table 2**, 0.1 is the optimal value of γ. Then, we adjust the remaining parameters in turn, and we can finally get optimal values of all the parameters as shown in **Table 3**.

**Table 3:** Optimal values of all parameters.

Parameters	Optimal value
*n_estimators*	350
*γ*	0.1
*min_child_weight*	1
*max_depth*	8
*subsample*	0.8
*colsample_bytree*	0.8
*learning_rate*	0.1

Using the optimal parameters in **Table 3**, the absolute error of CHAD on validation set is 0.1513 and is 0.1518 on test set. It can be seen that after the configuration of parameters, performance of the model was improved. Therefore, parameter adjustment is helpful for improving the accuracy.

In addition, XGBoost is highly interpretable. After the tree model is created, the importance score for each feature can be obtained directly. The importance scores are calculated and ranked for each feature in the dataset. In a single tree model, the importance score of each feature is calculated by the amount of improved performance measures for the split-point. The larger the improvement of a feature to the split point (closer to the root node), the more important the feature is.

**Figure 3:**
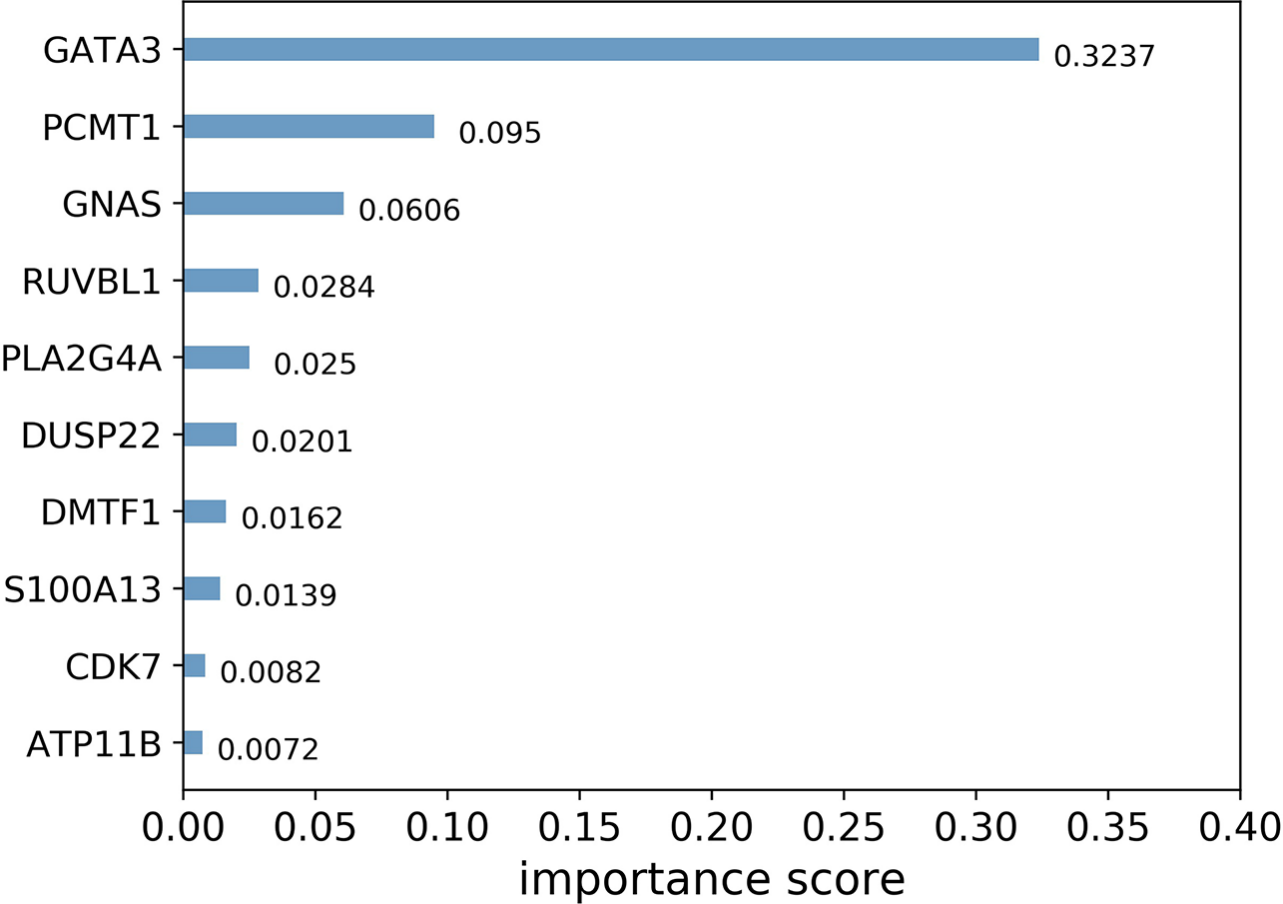
Top 10 landmark genes with the highest importance scores in the CHAD gene expression prediction task and their specific scores.

In general, importance scores measure the value of features in tree model construction. **Figure 3** shows the top 10 landmark genes with the highest importance scores in the CHAD gene expression prediction task and their specific scores. It can be seen that three landmark genes: GATA3, PCMT1, and GNAS score the highest in the prediction task, which also suggests that these three genes are the key genes in the prediction of CHAD gene expression value.

### Performance Comparison

#### Performance on GEO Data

In the experiment, we trained six models: LR, LR-L1, LR-L2, KNN, D-GEX, and XGBoost, respectively on the training set, and optimized parameters according to the performance on the validation set. Finally, we evaluated the prediction ability of various models according to their performance on the test set.

For each target gene *t*, we define the Mean Absolute Error as follows:

(8) $$\mathrm{MA}E_{\left(t\right)}=\frac{1}{N}\sum_{i=1}^{N}\left|y_{i(t)}-{\hat{y}}_{i(t)}\right|$$

where *N* is the number of samples.

**Figure 4:**
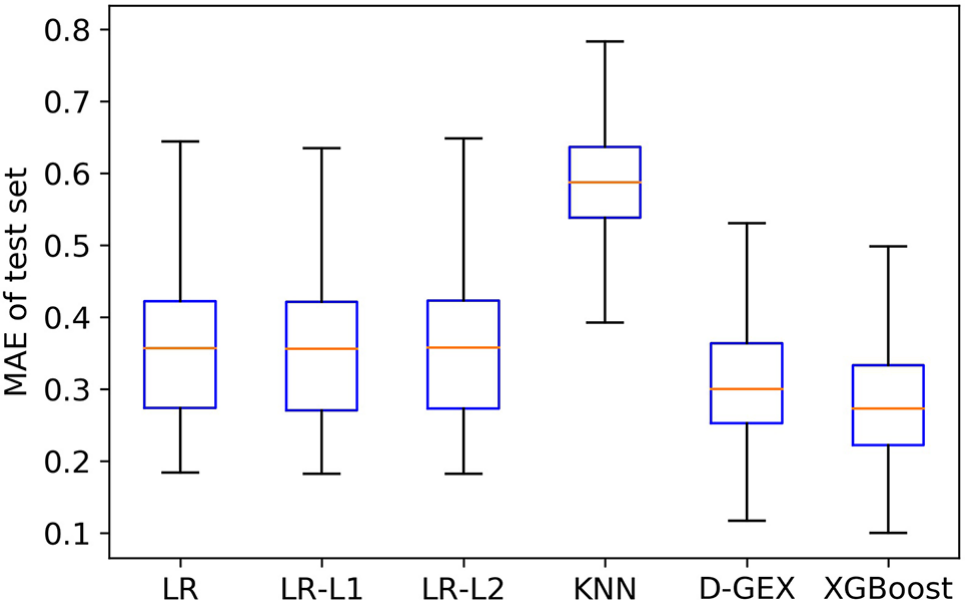
The Mean Absolute Error (MAE) distribution boxplot of the six algorithms on the test set.

**Figure 4** is the boxplot of MAE distribution of the predictive values of all the 9,520 target genes by six algorithms on the test set. As **Figure 4** shows, the XGBoost algorithm outperforms LR, LR-L1, LR-L2, and KNN significantly, and has a better distribution than D-GEX.

**Figure 5:**
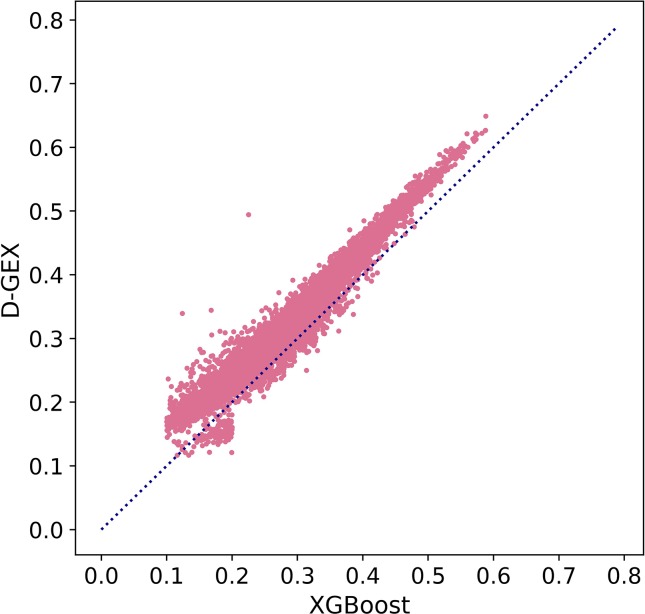
The Mean Absolute Error (MAE) score of each target gene predicted by XGBoost model compared with D-GEX on the test set. The x-axis is the MAE score of XGBoost model, and the y-axis is the MAE score of D-GEX.

Besides, we further explored MAE score in **Figure 5** to prove our conclusion. **Figure 5** showed the scatter plot of MAE of XGBoost compared with D-GEX on test set. Points above the diagonal indicated that the XGBoost model outperformed D-GEX on these target genes, and we found that the XGBoost model had a lower MAE than D-GEX on 91.5% of the entire set of target genes

In addition, we define overall error as follows, which represents the mean value of MAE on all target gene:

(9) $$\mathrm{overall}\;\mathrm{error}=\frac{1}{T}\sum_{t=1}^{T}\left[\frac{1}{N}\sum_{i=1}^{N}\left|y_{i}(t)-{\hat{y}}_{i}(t)\right|\right]$$

where N is the number of samples and T is the number of target genes.

**Table 4** shows the overall errors of six algorithms on validation set and test set. It can be seen that the results of XGBoost algorithm on both validation set and test set have achieved lower overall error, indicating that the XGBoost algorithm used in this paper has a good prediction ability and generalization ability for gene expression value prediction task.

**Table 4:** The overall error of six algorithms on validation set and test set.

Algorithm	*Overall error*	
	Validation set	Test set
LR	0.378	0.378
LR-L1	0.377	0.378
LR-L2	0.378	0.378
KNN	0.586	0.587
D-GEX	0.312	0.320
XGBoost	**0.280**	**0.282**

The figures in bold represent the best results on validation set and test set, respectively.

#### Performance on RNA-Seq Expression Data

To further study the practicality of XGBoost model in this task, we conducted a cross-platform experiment the same as Chen et al. (Chen et al., 2016). We used the training set of GEO data to train the models, and 1,000G expression data was used as validation set to tune parameters, and we finally evaluated the performance on the GTEx expression data. The results of all five models were shown in **Table 5**.

**Table 5:** The overall error of six algorithms on 1,000G data and GTEx data.

Algorithm	Overall error	
	1,000G data	GTEx data
LR	0.805	0.470
LR-L1	0.746	0.567
LR-L2	0.805	0.470
KNN	0.747	0.652
D-GEX	0.749	0.453
XGBoost	**0.733**	**0.439**

The figures in bold represent the best results on 1000G data and GTEx data, respectively.

The overall errors on the RNA-seq expression data further indicate the XGBoost model surpassed all the other learning models. Although for this specific task, the training set and the test set were generated from different platforms. This suggested that the XGBoost model performs well in this task and has a good generalization ability.

## Discussion

The gene expression value prediction algorithm based on XGBoost outperforms the D-GEX algorithm, and is better than the traditional machine learning algorithms such as Linear Regression and KNN.

In the task of predicting gene expression values, the number of landmark genes is large, which leads to the high dimensionality of input features. This makes the model very easy to fall into overfitting. For the deep network of D-GEX, not only the input dimension is very high, the output dimension is even higher. Therefore, it is difficult to train a very accurate model, and the processing of parameter adjustment is extremely complicated as well. Apart from this, poor interpretability is also a disadvantage of deep network.

In the XGBoost algorithm, the control of the complexity of the model is added. Random sampling of samples and features during training time makes the trained model less likely to overfit, which improves the generalization ability of the model, and eventually, the predictive errors for the validation set and test set are significantly reduced. Furthermore, XGBoost is more focused on the interpretability of the model, so we can learn which landmark genes have greater influence on the expression value of each target gene.

At the same time, although there is a serial relationship between trees in the XGBoost algorithm, the same level nodes can be parallelized, and the multi-threading of the CPU is automatically used for parallel computing, which makes the XGBoost model faster than traditional tree models, and the XGBoost model has a higher practical value.

## Data Availability Statement

All datasets generated and analyzed for this study are included in the article/**Supplementary Material**.

## Author Contributions

HZ conceived the research. WL, YY, HZ, and XQ designed the research. WL implemented the research. WL, HZ, and YY wrote the manuscript. All authors read and approved the final manuscript.

## Funding

This study is supported by the Major Program of the National Social Science Fund of China (Grant No. 18ZDA362).

## Conflict of Interest

The authors declare that the research was conducted in the absence of any commercial or financial relationships that could be construed as a potential conflict of interest.
