# Supplementary material for: Gene Expression Value Prediction Based on XGBoost Algorithm
Source: Front Genet. 2019 Nov 12;10:1077. doi: 10.3389/fgene.2019.01077 (PMC6861218; doi:10.3389/fgene.2019.01077)
Supplement: Supplementary file 1 [file DataSheet_1.docx]

Supplementary Material

# Details of XGBoost Algorithm:

The details of XGBoost algorithm is introduced as follows ([Chen T and Guestrin C, 2016](#r11xgboost)):

Integrate the tree model with addition method, assuming a total of K trees, and use *F* to represent the basic tree model, then:

$\hat{y}_{i}=\sum_{k=1}^{K} f_{k}(x_{i}),f_{k}\in F$ (1)

The objective function is:

$L=\sum_{i} l(\hat{y}_{i},y_{i})+\sum_{k} \Omega(f_{k})$ (2)

where $l$ is the loss function, which represents the error between the predictive value and the true value;$\Omega$ is the function used for regularization to prevent overfitting:

$\Omega(f)=\gamma T+\frac{1}{2}\lambda\left\|w\right\|^{2}$ (3)

where$T$represents the number of leaves per tree, and *w* represents the weight of the leaves of each tree.

${\hat{y}_{i}}^{(t)}$ represents the predictive value after the $t_{th}$iteration, then:

$\hat{y}_{i}^{(t)}=\hat{y}_{i}^{(t-1)}+f_{t}(x_{i})$ (4)

Therefore, the objective function can be expressed as:

$L^{(t)}=\sum_{i} l(\hat{y}_{i}^{(t-1)}+f_{t}(x_{i}),y_{i})+\Omega(f)$ (5)

The second-order Taylor expansion of the objective function is:

$L^{(t)}=\sum_{i} [l(y_{i},\hat{y}_{i}^{(t-1)}+g_{i}f_{t}(x_{i})+\frac{1}{2}h_{i}f_{t}^{2}(x_{i}))]+\Omega(f)$ (6)

where:

$g_{i}=\partial_{\hat{y}_{i}^{(t-1)}}l(y_{i},\hat{y}_{i}^{(t-1)})$ (7)

$h_{i}=\partial_{\hat{y}_{i}^{(t-1)}}^{2}l(y_{i},\hat{y}_{i}^{(t-1)})$ (8)

Ignore constant term, (6) can be written as:

$L^{(t)}=\sum_{i} \left[g_{i}f_{t}(x_{i})+\frac{1}{2}h_{i}f_{t}^{2}(x_{i})\right]+\Omega(f)$ (9)

Define $I_{j}=\left\{ i\left| q(x_{i})=j \right. \right\}$ as the $j_{th}$leaf node and (9) can be written as:

$\begin{matrix} L^{(t)}=\sum_{i} \left[g_{i}f_{t}(x_{i})+\frac{1}{2}h_{i}f_{t}^{2}(x_{i})\right]+\frac{1}{2}\lambda\sum_{j=1}^{T} w_{j}^{2} \\ \text{ }=\sum_{j=1}^{T} \left[\left(\sum_{i\in I_{j}} g_{i}\right)w_{j}+\frac{1}{2}(\sum_{i\in I_{j}} h_{i}+\lambda)w_{j}^{2}\right]+\gamma T \end{matrix}$ (10)

When the derivative of the objective function is equal to 0 , the optimal weight is:

$w_{j}^{*}\text{= - }\frac{\sum_{i\in I_{j}} g_{i}}{\sum_{i\in I_{j}} h_{i}+\lambda}$ (11)

When $w_{j}={w_{j}}^{*}$, the objective function is:

$\tilde{L}^{(t)}(q)=-\frac{1}{2}\sum_{j=1}^{T} \frac{(\sum_{i\in I_{j}} g_{i})^{2}}{\sum_{i\in I_{j}} h_{i}+\lambda}+\gamma T$ (12)

XGBoost uses a greedy algorithm instead of an enumeration method to improve computational efficiency. $I_{L}$represents the set of all left nodes after each split, and $I_{R}$ is the set of all right nodes after each split, then after each split, the information gain of the objective function is:

$Gain=\frac{1}{2}\left[\frac{(\sum_{i\in I_{L}} g_{i})^{2}}{\sum_{i\in I_{L}} h_{i}+\lambda}+\frac{(\sum_{i\in I_{R}} g_{i})^{2}}{\sum_{i\in I_{R}} h_{i}+\lambda}-\frac{(\sum_{i\in I} g_{i})^{2}}{\sum_{i\in I} h_{i}+\lambda}\right]-\gamma$ (13)

**Data**

The data used in our paper was kindly provided by the author of paper “Gene expression inference with deep learning”, and we don't have the ownership of the data. The readers who want to access the data can contact the author of this article.

**References:**

Chen, T., and Guestrin, C. (2016). Xgboost: A scalable tree boosting system. In *Proceedings of the 22nd acm sigkdd international conference on knowledge discovery and data mining* (pp. 785-794). ACM.[doi:10.1145/2939672.2939785](https://doi.org/10.1145/2939672.2939785)

Chen, Y., Li, Y., Narayan, R., Subramanian, A., and Xie, X. (2016). Gene expression inference with deep learning. *Bioinformatics*, *32*(12), 1832-1839. [doi: 10.1093/bioinformatics/btw074](https://doi.org/10.1093/bioinformatics/btw074)
